# To kill or not to kill – The role of the tumor microenvironment in shaping group 1 ILC functions

**DOI:** 10.1016/j.smim.2022.101670

**Published:** 2022-11

**Authors:** Nils Christian Müller, Chiara Romagnani

**Affiliations:** a Innate Immunity, German Rheumatism Research Center (DRFZ), Leibniz Association, Virchhowweg 12, 10117 Berlin, Germany; b Department for Gastroenterology, Infectious diseases, Rheumatology, Charité - Universitätsmedizin Berlin, corporate member of Freie Universität Berlin, Humboldt-Universität zu Berlin and Berlin Institute of Health, Campus Benjamin Franklin, Berlin, Germany

**Keywords:** NK cells, ILC1, Cancer, Tumor microenvironment, IL-15, TGF-β

## Abstract

Group 1 innate lymphoid cells (ILC) comprise two major IFN-γ producing populations, namely Natural Killer (NK) cells, and ILC1s. Recent studies have revealed a complex and diverse composition of group 1 ILC subsets infiltrating different tumors. In this review, we will outline the commonalities and differences between group 1 ILC subsets in both mice and humans, discuss how the tissue and tumor microenvironment shapes their phenotype and functions, as well as describe their contrasting roles in the response to different cancers.

## 1 Introduction

In the last 10–15 years, innate lymphoid cells (ILCs) have been characterized as the latest addition to the innate immune system. In contrast to their adaptive counterpart, ILCs rely on the expression of germline-encoded receptors sensing endogenous and environmental signals, spanning from cytokines and neuromediators to pathogens, microbiota, and nutrients [1]. Analogous to the T helper cell classification, ILCs are divided into three major subgroups based on their transcription factor and cytokine expression. Group 1 ILCs (g1 ILCs), consisting of natural killer (NK) cells and ILC1s, express T-bet and are major producers of IFNỵ, Group 2 ILCs (ILC2s) display high expression of GATA3, IL-4, and IL-13 and Group 3 ILCs, consisting of ILC3s and lymphoid tissue inducer (LTi) cells, that mainly express RORỵt and IL-22 [1].

A significant amount of literature has highlighted the role of ILCs during infections and inflammation; conversely, the functions displayed by different ILC subsets, especially ILC2 and ILC3, in the context of cancer are just starting to emerge [2]. Among g1 ILCs, the role of NK cells in anti-tumor responses and immunosurveillance has been extensively characterized [3]. However, most of these data were generated before the identification of ILC1s, both in mice and humans. As the distinction between ILC1s and NK cells can be challenging, especially within tissues, the question of which aspects of the anti-tumor response are mediated by ILC1s and which by NK cells remains open. In light of these considerations, this review’s focus is to guide through the ongoing debate on human and murine g1 ILC heterogeneity as well as give a critical overview of the proposed role of different g1 ILC subsets in cancer.

## 2 Group 1 ILCs in mice

Murine ILC1s and NK cells are both marked by the co-expression of NKp46, NK1.1, and T-bet and can be found throughout many tissues. ILC1s and NK cells are commonly distinguished by the expression of CD49a or CD200r1 associated with ILC1s, and of CD49b (DX5) and Eomes, typically displayed by NK cells [4], [5], [6], [7], [8], [9]. Another characteristic commonly used to separate ILC1s from NK cells is the fact that ILC1s are locally maintained as tissue-resident cells expressing the interleukin 7 receptor (CD127), while NK cells are mainly circulating, replenished by bone marrow-derived precursors, and maintained by interleukin 15 (IL-15) signaling [5], [10], [11].

Recently, McFarland et al. applied single-cell RNA-Sequencing (scRNA-Seq) on NKp46^+^ NK1.1^+^ g1 ILCs isolated from various organs, including two experimental tumor settings, allowing for the first time a systematic comparison of the transcriptional states of g1 ILCs from different tissues. This study revealed a common signature for circulating NK cells, marked by *Eomes, Sell,* and *Klf2,* typically associated with cells from the spleen, blood and lymph nodes (LN), and to a lesser extent in cells from the liver and visceral adipose tissue (VAT) [12]. Superimposed on this common signature, circulating NK cells display a gradient of gene expression, highlighted by differential expression of *Cd27* and *Itgam* (CD11b) and confirming the standing definitions of NK cell maturation states [12], [13], [14]. Elevated *Tcf7* (TCF-1) transcripts, along with *Ly6e, Cd7, Xcl1, Cxcr6, Il7r*, and *Ikzf2* are associated with high CD27 surface expression, while low *Tcf7* and high *Klrg1, Ly6c2, Gzma, Ccl5, Zeb2, Irf8,* and *Klf2* transcripts reflect a mature effector program correlating with low CD27 and high CD11b protein expression [12]. During maturation from the CD27^high^CD11b^low^ to the CD27^low^CD11b^high^ stage, NK cells acquire stronger cytotoxicity, while immature NK cells produce higher amounts of cytokines [12], [13], [14].

Besides circulating NK cells, clusters of tissue-associated NK1.1^+^NKp46^+^ cells were identified, sharing a transcriptional signature centered around *Zfp683* (Hobit) and the key ILC transcription factors (TFs) *Id2, Rora, Ahr,* and *Ikzf3,* along with low to no *Eomes* and *Tcf7* (TCF-1) expression, preferentially marking tissue-resident ILC1 [12], [15], [16]. However, considerable similarities remain between the signature displayed by circulating immature CD27^high^CD11b^low^ NK cells and the one of tissue-associated NK1.1^+^ NKp46^+^ cells, suggesting that the line between ILC1s and NK cells might be blurry. Whether these similarities stem from a common developmental relationship or indicate a similar maturation state remains to be elucidated.

Furthermore, g1 ILCs found in salivary glands (SG) and uterus co-express Eomes, CD49b, and CD49a and share many transcripts with circulating CD27^high^CD11b^low^ NK cells [6], [8], [12], [17], [18], [19], [20]. These cells display tissue residency properties similar to ILC1, as demonstrated in parabiosis experiments [8], [10], [20], [21]. This intermediate phenotype of SG CD49a^+^ CD49b^+^ g1 ILCs is a result of TGF-β imprinting as the abrogation of TGF-β receptor 2 (TGF-β-R2) signaling on NKp46^+^ cells substantially reduces SG g1 ILCs. The decrease in numbers is paralleled by the acquisition of NK maturation markers and loss of CD103, CD69, and CD39 [21]. Interestingly, the SG g1 ILC phenotype induction seems to occur via a non-canonical SMAD4-independent TGF-β pathway that includes JNK, as SG g1 ILC numbers and phenotype are not affected in *Smad4*^*fl/fl*^ x *Ncr1*^*iCre*^ mice [21], [22].

Further underlining the importance of TGF-β signaling in shaping g1 ILC phenotype is the surprising observation that *Smad4*-deficient NK cells switch to an ILC1-like phenotype throughout all tissues, as indicated by upregulation of CD49a. This phenomenon seems independent of TGF-β-R2, but dependent on TGF-β-R1, suggesting it might act via a different non-canonical SMAD4-dependent TGF-β pathway than the one inducing the SG g1 ILC phenotype [21], [23]. As the uterus, alike the SG, is characterized by a TGF-β-rich microenvironment, it is plausible that similar pathways might favor the local accumulation of uterine CD49a^+^ CD49b^+^ g1 ILCs [19]. This data demonstrates that even CD49b, CD49a, and CD200R1 cannot be considered universal surface markers that unequivocally distinguish NK cells and ILC1s throughout all tissues [6], [8], [10], [12], [18], [19], [20].

The struggle in assigning g1 ILC identity in certain tissues highlights the importance of the local microenvironment in shaping their phenotype. Besides SG and uterine g1 ILCs, ILC1s and NK cells from the small intestine (SI) lamina propria display pronounced tissue-imprinting and have been proposed to be transcriptionally closer related to SI LP NKp46^+^ ILC3 than to liver r splenic g1 ILCs [24]. However, this can also be related to the observation that a consistent fraction of SI LP NKp46^+^NK1.1^+^ cells is composed of “ex-ILC3s”, i.e. ILC3s that have lost RORỵt expression and appear phenotypically indistinguishable from ILC1s [5], [12], [25]. Indeed, plastic conversion of other ILC lineages towards g1 ILCs as a result of local signals might also contribute to determine g1 ILC signature heterogeneity across tissues.

The importance of the tissue microenvironment in shaping g1 ILCs is further emphasized by the intestinal intraepithelial lymphocyte (IEL) compartment. In 2013, Fuchs et al. identified a population of intraepithelial T-bet^+^ ILC1 (ieILC1) that, in contrast to SI LP ILC1, lack CD127, express low levels of Eomes, and have cytotoxic capabilities [26]. Interestingly, in addition to the ieILC1, other two T-bet expressing innate cell populations have been described within the SI IEL compartment. The first population was identified by intracellular CD3γ (iCD3γ) and surface expression of CD103. A fraction of these cells additionally expresses CD8α, similar to IEL T cells. Remarkably, while being Eomes^-^, iCD3γ^+^ innate IEL express high levels of Granzyme (Gzm) A and B [27], [28]. The second T-bet^+^ innate IEL population is marked by the expression of Ly49E and, in contrast to the iCD3γ^+^ innate IEL, expresses GzmB together with Eomes. Nonetheless, Ly49E^+^ innate IEL seem to display low cytotoxic activity and do not kill YAC-1 cells [29]. As both iCD3γ^+^ and Ly49E^+^ innate IEL subsets lack NKp46 and NK1.1 expression, they do not fit into the common definition of g1 ILCs and have not been included in the systematic scRNA-Seq analysis of g1 ILCs performed by McFarland et al. [12], [27], [29]. However, as we will discuss further, the relationship between these cells and g1 ILCs remains to be ascertained.

Heterogeneity among the ILC1 compartment was recently defined in the liver, where Eomes^-^CD49a^+^ ILC1s were subdivided based on the expression of CD160 and GzmA. It was shown that CD160^+^ GzmA^-^ ILC1 produce higher levels of cytokines, while CD160^-^ GzmA^+^ ILC1 display higher cytotoxicity toward YAC-1 cells [30]. Using a newly generated double reporter mouse, CD49a^+^ CD49b^-/+^ ILC1s from the liver and SG were further dissected according to history (fate map, FM) or actual expression of GzmC into GzmC^+^ GzmC^FM+^, GzmC^-^ GzmC^FM+^ and GzmC ^-^GzmC^FM-^ cells [24], [31], [32]. Based on the expression of genes related to cell cycle and phenotype, the authors suggested that GzmC^-^ GzmC^FM-^ cells might be a precursor population of the fully mature GzmC^+^ GzmC^FM+^ effector cells [32]. Yet another approach to stratify liver Eomes^-^ CD49a^+^ ILC1s was proposed by Friedrich et al.*,* based on the expression levels of CD127. Liver CD127^+^ ILC1 cells produced higher levels of cytokines, exhibiting a “helper-like” phenotype, while CD127^-^ ILC1s, which are additionally marked by expression of iCD3γ and high levels of Gzm A, B, and C, display a killer-like phenotype. These findings were in line with the distinction based on CD160 and GzmA by Di Censo et al., as CD127^-^ ILC1s exhibit lower CD160 expression [30], [33]. Keeping with the idea that GzmC expression marks a fully matured ILC1 phenotype, Friedrich et al. propose that heterogeneity within liver ILC1s follows a trajectory of Hobit-dependent differentiation from CD127^+^ towards CD127^-^ iCD3γ^+^ Gzm^+^ ILC1s [15], [32], [33]. Interestingly, a similar gene expression gradient from CD127^+^ helper towards CD127^-^ cytotoxic ILC1s can also be observed from SI LP ILC1s to the ieILC1s sitting in the IEL compartment, raising the question of whether these cells might be developmentally related and followed a similar trajectory [5], [26].

Further heterogeneity of liver ILC1s was shown in a recent study that used the marker Ly49E to separate liver ILC1s into embryo-derived Ly49E^+^, which are a subset of the cytotoxic iCD3γ^+^ ILC1s, and postnatally occurring Ly49E^-^ ILC1s. Expression of Ly49E is stable over time, with no observed conversion of Ly49E^-^ ILC1s into Ly49E^+^ ILC1s [34]. Of note, some of the markers used to distinguish these liver ILC1 subsets, namely iCD3γ and Ly49E, are the same ones that define the T-bet^+^ NKp46^-^ NK1.1^-^ innate IEL populations, hinting towards a broader and still unexplored role of these molecules for innate type 1 immunity.

In conclusion, g1 ILCs in mice span a diverse spectrum of phenotypes ranging from classical helper ILC1s to cytotoxic NK cells and include several intermediates that differ in cytokine production and killing potential. This heterogeneity is possibly reflecting distinct maturation stages and/or the influence of the different tissue microenvironments.

## 3 Group 1 ILCs in humans

In humans, the g1 ILCs are present in equally or even more varied forms as in mice. Circulating NK cells are historically identified as Lin^-^CD56^+^ cells, and are separated into cytokine-producing CD56^bright^CD16^-^ and cytotoxic CD56^dim^CD16^+^ [35]. These subsets, which were proposed to be developmentally related, are distinguished by high expression and promoter accessibility of *RUNX2*, *BACH2, GATA3,* and *TCF7* in immature CD56^bright^ NK cells, and of *ZBTB16*, *BCL11B, PRDM1* (encoding Blimp-1) and *ZEB2* in more terminally differentiated CD56^dim^, resembling the different maturation stages of murine NK cells [12], [14], [36], [37], [38], [39], [40]. In addition to this, NK cells have been described in most organs, where they seem to exhibit a more immature phenotype, resembling CD56^bright^ NK cells [41]. However, cells identified as CD56^+^CD16^-^ do not exclusively contain NK cells, as CD56 is also expressed by a subset of ILC3s, as well as on a subset of circulating ILC progenitors (ILCPs) with group 1 and group 3 ILC lineage commitment [42], [43], [44], [45], [46]. Similarly, the human correspondents of murine ieILC1s identified in tonsils and within SI IEL by Fuchs et al., express CD56 together with low levels of Eomes. This, together with the lack of CD127 expression, makes it difficult to distinguish human NK cells and ieILC1s [26]. Accordingly, tonsil-derived ieILC1 share the expression of many canonical NK cell genes and in the direct comparison, only a few differentially expressed genes emerge, including *ITGAE* (CD103), *NCR2* (NKp44), and *IL23R* [38], [47].

In line with the murine data, another ILC1 population with a helper-phenotype lacking cytotoxic molecules was also described in humans. These cells, originally identified in human tonsil and SI LP, are marked by the expression of the common ILC markers CD127 and CD161 and defined by the lack of CD56 and other NK/ILC2/ILC3 surface markers, including CD94, NKp44, c-Kit, and CRTH2 [48]. However, the existence of CD127^+^ ILC1 as a separate lineage has been openly questioned, and it was suggested that these cells are T cell contaminants [49]. This notion seems to be corroborated by a scRNA-Seq study of human ILCs from different tissues that revealed clustering of ILC1s together with T cells, along with enrichment of T cell-associated genes and TCR rearrangements [50]. Another study, assaying CD127^+^ ILCs and CD94^+^ NK cells isolated from the *lamina propria* of a Crohn’s disease patient was also unable to identify a distinct cluster of CD127^+^ ILC1 but instead found a population of cytotoxic CD127^+^CD94^+^ ILC1-like cells. These cells, which share several features with peripheral blood CD56^bright^ NK cells, uniquely displayed high expression of Granulysin, and further increase the complexity of human g1 ILCs [51].

In analogy to what we have described in mice, the separation of ILC1s and NK cells in tissues might be aggravated by effects imposed on human g1 ILCs by the different tissue microenvironments. This is exemplified by decidual NK cells (dNK), which include three different CD56^bright^ subsets with a strong TGF-β signature, similar to their murine counterparts [6], [52]. One of these subsets expresses CD103 and, despite being NKp44^-^, appears quite similar to intestinal or tonsillar ieILC1 [6], [26], [52]. Moreover, “ex-ILC2” or “ex-ILC3” displaying a g1 ILC phenotype have been described in humans, possibly further contributing to g1 ILC heterogeneity in tissues [47], [53], [54], [55].

Taken together, the substantial similarity of the transcriptome between ieILC1s and NK cells and the lack of a clear transcriptional signature for CD127^+^ ILC1s make the distinction of specific human g1 ILC subsets a recurring problem.

## 4 Anti-tumor functions of group 1 ILCs are inhibited by the tumor microenvironment

NK cells display potent anti-tumor activity. They detect cancer cells via two different mechanisms, the recognition of “missing self” as well as “induced self”. The recognition of “missing self” describes the lack of signaling from inhibitory NK cell receptors (NKR), like NKG2A and inhibitory killer immunoglobulin-like receptors (KIRs) in humans or Ly49 receptors in mice, which bind proteins constitutively expressed by healthy cells, like MHC-I [56]. These proteins are commonly downregulated by cancer cells as a means of immune evasion [57]. In contrast, the recognition of “induced self” works via activating NKRs, like NKG2D, which binds self ligands up-regulated as a consequence of dysregulated proliferation, DNA damage, endoplasmic reticulum (ER) stress, and other cell states associated with malignant cell transformation [56]. The collective input of both inhibitory and activating NKRs results in a fine-tuned response ensuring the selective killing of target cells, through the directed release of cytotoxic granules or the engagement of death receptors on the cell surface [56], [58]. Additionally, NK cell activation leads to the release of large amounts of IFNγ, TNF, and chemokines that exert strong anti-tumor functions and recruit immune cells into the tumor [56].

Early experiments demonstrating a protective role of NK cells against solid tumors used anti-NK1.1 or anti-asialoGM1 antibodies to deplete NK cells in methylcholanthrene (MCA)-induced fibrosarcoma and transplantable tumor models [59], [60]. Later studies employed *Ncr1*^*-/-*^ mice to show that NKp46 is involved in the prevention of tumor metastasis [61]. Notably, both NK1.1 and NKp46 are not only expressed by NK cells, but also by ILC1s and by a subset of ILC3s. Therefore, the anti-tumor functions ascribed to NKp46^+^ cells are not necessarily exhibited by NK cells alone. Recent reports have attempted to distinguish between effects mediated by ILC1s and by NK cells, both of which can produce large amounts of cytokines and exert direct cytotoxicity against tumors.

One of the first studies to address this issue analyzed Lin^-^ CD45^+^ NK1.1^+^ NKp46^+^ cells infiltrating the MCA-induced fibrosarcoma cell line MCA1956. Besides classical CD49a^+^ CD49b^-^ ILC1s and CD49a^-^ CD49b^+^ NK cells, the authors observed a third population co-expressing CD49a and CD49b and displaying an intermediate expression of ILC1 and NK signature genes, therefore termed “intermediate ILC1” (intILC1) [62]. These intILC1s closely resemble tissue-resident SG g1 ILCs, which also co-express CD49a and CD49b. As already described for SG g1 ILCs, the conversion of tumor-infiltrating NK cells towards intILC1s was also shown to be dependent on TGF-β-R2 signaling [21], [62], indicating that the tumor microenvironment (TME) can alter the phenotype of infiltrating NK cells through the release of TGF-β, further promoting the expression of inhibitory receptors and exhaustion markers, like NKG2A, CTLA-4, and LAG-3. Therefore, it was proposed that intILC1 might represent some form of exhausted NK cells [63]. Additionally, the strong similarity between intILC1s and SG g1 ILCs hints toward a role for TGF-β in promoting the expression of CD39 and CD73 on intILC1s, as previously demonstrated for protumorigenic myeloid cells [21], [62], [64]. CD39 and CD73 up-regulation would enable them to sequester extracellular pro-inflammatory ATP into anti-inflammatory adenosine and in turn dampen anti-tumor NK cell function even further. Supporting the notion that NK cells exposed to a TGF-β-rich TME develop an ILC1-like state and display decreased anti-tumor functionality is the observation that NKp46^+^ cells from *Smad4*^*fl/fl*^ x *Ncr1*^*iCre*^ mice acquire ILC1 characteristics via a non-canonical SMAD4-dependent, but TGF-β-R2 independent, pathway and lose the ability to protect from lung metastases in the B16 melanoma model [23].

Further confirmation that NK cells might convert to intILC1 inside the TME was presented by the study by McFarland et al. in 2021, in which scRNA-Seq of g1 ILCs from PyMT breast cancer and B16F10 melanoma was performed. This data revealed an increased frequency of CD49a^+^ CD49b^+^ cells co-expressing *Irf8*, a marker of circulating NK cells, and low levels of *Gzma,* suggesting that the TME can hamper the cytotoxic functions of infiltrating NK cells through their conversion into intILC1s [12], [62]. Additionally, these cells showed reduced levels of *Ccl5*, which together with XCL1, attracts XCR1^+^ conventional dendritic cells 1 (cDC1) into the TME, thereby promoting tumor control [12], [65]. Inside the TME, Prostaglandin E2 (PGE2) inhibits CCL5 and XCL1 production and leads to reduced NK cell viability, highlighting that the TME can interfere with NK cell-mediated anti-tumor functions through multiple mechanisms [65]. However, it should be noted that in the study from McFarland et al.*,* only a reduction in *Ccl5,* but not in *Xcl1,* was observed, suggesting that NK cells might still be able to attract cDC1 [12], [65].

Another indicator that NK cell conversion toward intILC1s is associated with impaired anti-tumor immune responses comes from a study that generated multiple MCA tumor cell lines in NKp46-depleted mice. These tumor lines showed a graded sensitivity toward NK1.1 depletion. Intriguingly, the authors could show that the amount of ILC1s and intILC1s within the different tumors inversely correlate with their sensitivity towards NK1.1 depletion, arguing for a negative role of ILC1s in tumor protection [66]. This finding was confirmed using B16F10 and MC38 cell lines. B16F10 tumors, which are controlled by NK cell functions, were infiltrated almost exclusively by CD49a^-^CD49b^+^ NK cells, while MC38 tumors had high frequencies of ILC1s and intILC1s. Not only were MC38 tumors “insensitive” to NK1.1 depletion, but NK1.1 treatment even improved the survival of tumor-bearing mice [66]. Together, these observations argue for an active role of the TME in modulating g1 ILC mediated anti-tumor responses, where TGF-β producing tumors convert NK cells into less cytotoxic ILC1s with impaired anti-tumor functions.

Additional evidence for the role of the TME in thwarting the protective functions of g1 ILCs was obtained using intradermal injections of the mSCC38 cell line and through a two-stage model of chemically induced cutaneous squamous cell carcinoma (cSCC). In this model, skin carcinogenesis is initiated by the chemical mutagen 7,12-Dimethylbenzanthracene (DMBA) and tumor formation is promoted through phorbol 12-myristate 13-acetate (PMA, a.k.a. 12-O-Tetradecanoylphorbol-13-acetate (TPA)). As a result, benign skin papillomas are generated that can progress into malignant cSCC, with progressive chromosomal abnormality and aneuploidy [67], [68]. Transcriptomic analysis of CD3^-^ NKp46^+^ cells isolated from healthy skin and tumor revealed that helper ILC1s were enriched in papillomas, while NK cells were enriched in malignant tumors, indicating differential recruitment of g1 ILC populations. The enrichment of ILC1s in the papilloma stage might be explained by a sustained TGF-β exposure of tissue-resident g1 ILCs in the TME, while the conversion to the malignant stage and the associated additional mutations leads to an infiltration of NK cells from the periphery [68]. Despite the differential composition of g1 ILCs in the different tumor stages, all intratumoral g1 ILCs produced lower levels of IFNγ and presented increased expression of the exhaustion markers *Tigit, Pdcd1* (PD1), and *Lag-3,* the latter two being the most prominent in ILC1s [68]. While the authors did not distinguish between CD49a^-^ CD49b^+^ cells and CD49a^+^ CD49b^+^, leaving the possibility that part of the intratumoral Eomes^+^ cells are double-positive intILC1s, these findings support the notion that g1 ILC responses can be impaired when exposed to a defined TME [62], [68].

**Fig. 1:**
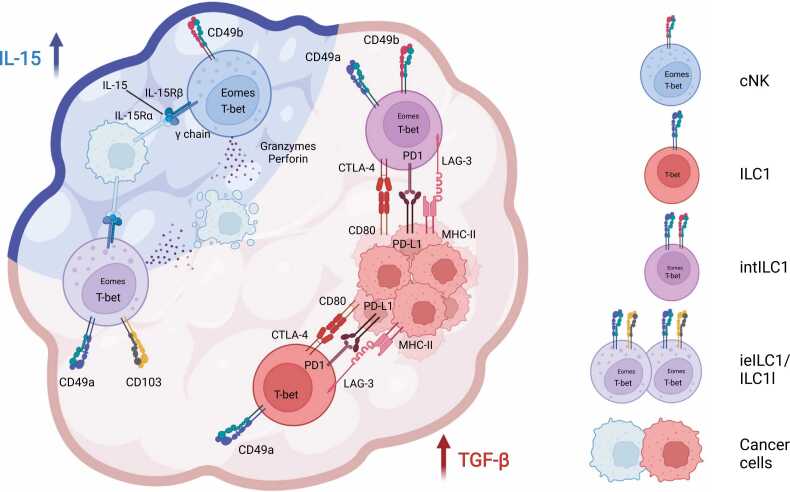
**Group 1 ILCs in the tumor microenvironment.** The tumor microenvironment (TME) shapes the phenotype of g1 ILCs. IL-15-producing tumors host CD49a^-^ CD49b^+^ conventional NK cells (cNK), while in tumors producing both IL-15 and TGF β, CD49a^+^ CD103^+^ intraepithelial ILC1 (ieILC1s) and ILC1-like cells (ILC1ls) can also be found. Both cNK and ieILC1s/ILC1ls can actively kill cancer cells via lytic granules containing granzymes and perforin. Tumors enriched in TGF-β, possibly without IL-15, promote intratumoral accumulation of CD49a^+^ CD49b^+^ Eomes^+^ intermediate ILC1s (intILC1s) and CD49a^+^ CD49b^-^ Eomes^-^ ILC1s. IntILC1s and ILC1s upregulate inhibitory and exhaustion receptors that bind to ligands on cancer cells and can lead to cancer immunoevasion.

Summed up, in several different solid tumor models, the TME can shift the g1 ILC phenotype away from cytotoxicity towards increased expression of exhaustion markers, effectively subverting their anti-tumor functions (Fig. 1).

## 5 ILC1s can yet kill cancer cells

In disagreement with the reports mentioned in Section 4, a series of studies have portrayed strong tumor-killing functions of ILC1s. In 2016, Dadi et al. exposed for the first time ILC1 cytotoxic capability in the context of cancer. Using the MMTV-PyMT tumor model, the authors showed infiltration of precancerous lesions and tumors by three different populations with cytotoxic properties, TCRβ^+^ T cells, TCRδ^+^ T cells, and TCR^-^ NK1.1^+^ NKp46^+^ innate lymphocytes, all of which express GzmB and share a similar transcriptome. Due to their innate properties, the infiltrating T cells were termed “innate-like T cells with high cytotoxic potential” (ILTCK) [31], [69]. The identified TCR^-^ NK1.1^+^ NKp46^+^ innate lymphocyte population, referred to as type 1-like ILCs (ILC1ls) in this study, differs from intILC1 described by Gao et al., as they express CD49a and CD103 while being largely negative for CD49b [31], [62]. ILC1ls, as well as ILTCKs, are tissue-resident cells that expand locally during tumor transformation and efficiently kill tumor cells using lytic granules [31]. Additionally, ILC1ls are CD127^-^ and express only low levels of the ILC1-signature cytokines IFNγ and TNF. These characteristics resemble the phenotype of fully differentiated effector ILC1s described by Friedrich et al. [31], [33]. Of note, around 10 % of the ILC1ls inside the tumor express Ly49E, indicating the possibility that the ILC1l population could consist of neonatal Ly49E^+^ and postnatal Ly49E^-^ cells, as recently identified in the liver [31], [34]. Moreover, ILC1ls express low levels of Eomes and *Cd160* and require IL-15 but not Nfil3 for their development, leaving ILC1l lineage identity still unresolved [31]. In a follow-up study, the same group attempted to address this question through further analysis of PyMT tumors and revealed that the GzmC^+^ ILC1ls consist of equal proportions of CXCR6^+^ and Eomes^+^ cells. Using adoptive transfer of Lin^–^Sca1^+^c-kit^+^ cells (LSK) from S1PR5-^FM^ mice into PyMT mice, the authors could show that none of the CXCR6^+^ ILC1ls and only about 25 % of the Eomes^+^ cells were S1PR5-^FM+^, indicating that the vast majority of intratumoral ILC1ls does not derive from NK cells. [32]. However, since some Eomes^+^ CD49a^+^ ILC1s were S1PR5^FM+^ and S1PR5 is only expressed by mature NK cells, but not by CD27^+^ CD11b^-^ immature NK cells, the question of whether cytotoxic ILC1ls can also be derived from NK cells remains open for debate [32]. This question is especially relevant since, as mentioned in Section 4, McFarland et al. showed in their study that tumor-specific clusters generated from the PyMT tumor model expressed *Irf8*, a marker of conventional NK cells, together with *Gzmc* [12].

Following this line of thought, GzmC^+^ ILC1ls are dependent on TGF-β for their development, just like the NK cell-derived intILC1s. Gzmc^ΔTgfbr2^ PyMT mice have significantly reduced numbers of GzmC^+^ cells in tumors along with accelerated tumor growth. These results are in direct conflict with the ones obtained in *Tgfbr2*^*fl/fl*^ x *Ncr1*^*iCre*^ mice, which showed improved survival in the MCA tumor model [32], [62]. This discrepancy might be explained by an additional role of IL-15, as suggested by in vitro culture experiments performed in the presence of IL-15 that equipped liver ILC1s with perforin-dependent cytotoxicity, regardless of GzmC expression [32]. Moreover, IL-15 deficiency in the tumor epithelium greatly diminished the amount of ILC1ls and accelerated PyMT tumor growth. Conversely, IL-15 from other sources, including dendritic, stromal, and endothelial cells, did not seem to affect intratumoral g1 ILCs, strongly suggesting that IL-15 trans-presentation by cancer cells might contribute to directly activating ILC1ls and promoting their phenotype [70].

The opposing instructing effects of the TME on g1 ILCs, resulting in either inhibition or activation of their anti-tumor functions is perhaps best demonstrated by a recent study by Ducimetière et al. that investigates the occurrence of liver metastases using either Lewis lung carcinoma cells (LLC) or MC38 colon carcinoma cells. By performing timed depletion experiments, the authors could show that ILC1s were necessary early on to inhibit metastatic seeding of both cancer lines, while NK cells had anti-metastatic functions early on but were also capable to combat late-stage tumor nodules in MC38 injected mice. Analysis of g1 ILCs revealed differences in frequency and cluster distribution between the two tumors. NKp46^+^ cells were reduced in LLC as compared to MC38 tumor nodules, suggesting that LLC cancers may inhibit g1 ILCs infiltration [71]. Moreover, LLC tumors presented a prominent cluster of CD49a^-^ Eomes^-^ infiltrating cells, with signatures compatible with immaturity or exhaustion. In contrast, MC38 tumors were enriched in CD49a^+^ Eomes^+^ cells expressing high levels of GzmB, closely resembling the ILC1l subset described in the PyMT tumor model. Enrichment for TGF-β and IL-2/IL-15 signaling pathway genes further underlined the similarities of CD49a^+^ Eomes^+^ cells to cytotoxic ILC1ls, which can exert potent anti-tumor functions [31], [71].

Notably, the CD49a^+^ Eomes^+^ cell cluster derived from MC38 tumors was enriched in glycolytic signatures, similar to what was observed by McFarland et al. in g1 ILCs from PyMT and B16F10 tumors [12], [71]. The importance of the glycolysis pathway for tumor suppression of NKp46^+^ cells was validated in *Ldha*^*fl/fl*^ x *Ncr1*^*iCre*^ mice that showed significantly more lung metastases after injection of B16F10 tumor cells [12]. Since hypoxia together with IL-15 can induce glycolysis in NK cells, this might be another important aspect explaining how IL-15 in the TME promotes anti-tumor responses of g1 ILCs [72].

Another indicator of the importance of the TME in shaping g1 ILC immune responses comes from experiments where B16F10 were peritumoral injected with CpG, thereby artificially changing the TME. G1 ILCs from these manipulated tumors exhibited higher amounts *of Prf1, Gzma,* and *Gzmb* transcripts, hinting towards a beneficial role of pro-inflammatory signals in counteracting TME immunosuppressive effects. However, due to the similarities of different g1 ILC populations, it remains challenging to assess whether the treatment diverted the differentiation of intILC1s into ILC1ls or rather prevented the conversion of NK cells to intILC1s [12].

Collectively, it is becoming evident that the TME can shape the phenotype of infiltrating g1 ILCs, leading to a variety of different fates. G1 ILCs can either display an exhaustion-like phenotype that potentially amplifies an immunosuppressive TME or can become highly efficient killers of cancer cells, essential for tumor clearance (Fig. 1).

## 6 Human group 1 ILCs in cancer

Due to a lack of unifying markers of g1 ILCs, such as NKp46, studies in human cancers seldom address them as a whole, but rather focus on all helper ILCs or NK cell subsets. Nonetheless, we will describe the most recent reports about human g1 ILCs in cancer and draw parallels to the two most common modes observed in murine g1 ILC responses in cancers – exhaustion versus cytotoxicity.

Data supporting the idea that the TME induces exhaustion of human g1 ILCs were generated by analyzing tumor samples from hepatocellular carcinoma (HCC) patients. scRNA-Seq analysis of Lin^-^ CD127^+^ CD56^-^ helper ILCs and Lin^-^ CD127^-^ CD56^+^ NK cells from the tumor and paired non-tumor tissue revealed four different g1 ILC clusters (collectively referred to as ILC1 by the authors), all of which expressed *EOMES* and other typical NK genes, including *GZMB, PRF1,* and *CCL5* [73]. The study went on to show that, among the four g1 ILC clusters, the one most enriched in the tumor also displayed the highest expression of inhibitory receptors/exhaustion markers, such as *KLRC1* (NKG2A) and *LAG3*, mirroring the phenotype of murine intILC1 in cancer [62], [73]. This was in line with an increased expression of TGF-β in HCC, as well as a correlation of high *TGFB2* expression with poor survival [74]. A similar phenotype was observed in tumor-infiltrating CD3^-^CD56^+^ g1 ILCs from breast cancer and sarcoma patients, revealing co-expression of multiple inhibitory receptors with CD73 [75].

Additional evidence for the immunosuppressive effects of the TME on human g1 ILCs stems from melanoma patients. While the overall number of ILCs in melanoma patient peripheral blood (PB) was lower than in controls, the percentage of ILC1s (gated as Lin^-^ CD127^+^ cKit^-^ CRTH2^-^) among ILCs, increased in PB as well as in tumor-infiltrated lymph nodes (TILN). It is important to note that no NK cell markers were included within the Lin staining; therefore, CD127^low^ CD56^bright^ NK cells were likely part of the ILC gate. Nonetheless, g1 ILCs from the TILN showed reduced IFNγ production upon restimulation, indicating a repressive influence from the tumor [76].

In another interesting study, scRNA-Seq analysis of Lin^-^ CD127^+^ CD94^-^ helper ILCs isolated from colorectal cancer (CRC) tissue samples identified the presence of an ILC1 cluster, also found in healthy mucosa, and a CRC tissue-specific ILC1-like (ctILC1-like) cluster. These ctILC1-like cells were marked by the expression of inhibitory/exhaustion molecules *TIGIT* and *CTLA4* as well as *CD27*, while the ILC1 cluster was enriched in cytolytic genes. This suggests a possible bifurcation among the g1 ILCs within the TME, where some become exhausted and others develop increased cytotoxicity, as observed in murine tumor models [77]. A cytotoxic g1 ILC population infiltrating CRC was also identified by de Vries et al., who used mass cytometry to show that 80 % of the CRC innate lymphocyte compartment, including helper ILCs and NK cells, are CD127^-^CD56^+^CD45RO^+^ cells. These cells showed high levels of cytotoxicity gene transcripts and of GzmB/Perforin proteins without restimulation, similar to the memory CD8^+^ T cell compartment from the same samples [78]. Additionally, the CD127^-^ CD56^+^ CD45RO^+^ cells expressed CD103 and CD69, potentially marking them as ieILC1s, in line with their intraepithelial localization [26], [78].

The importance of g1 ILCs in CRC is further underlined by data showing a proportional increase of CD127^+^ ILC1, accompanied by a decrease of ILC3s in the CRC tumor tissue, in comparison to adjacent non-affected tissue [53], [79]. It has been proposed that the ILC1 increase observed in CRC might result from conversion of ILC3, as previously shown in inflammatory bowel disease (IBD) [47], [53], [55]. Indeed Goc et al. observed that ILC3s inside the tumor tissue expressed higher levels of ILC1-related and ILC3-ILC1 transitional genes. However, the ILC1 transitional signature used, including cytotoxicity genes *PRF1, GZMB,* and *GNLY,* is associated to ieILC1s rather than CD127^+^ ILC1 [47], [53], leaving the possibility that in CRC ILC3s may convert into ieILC1s. This scenario would be consistent with the numerical increase of ieILC1s and the net decrease of all CD127^+^ helper ILCs in CRC tumors [53], [78], [79]*.* However, it is also possible that the increase of g1 ILCs in CRC is at least in part a consequence of plastic ILC2s converting into g1 ILCs under the influence of IL-12 [54]. Similarly, circulating CD7^+^CD127^+^CD117^+^ ILCPs, could infiltrate the TME and locally differentiate into g1 ILCs, thereby leading to the enrichment of g1 ILCs in the tumor tissue [45], [46].

Additional support for the importance of cells with an ieILC1 phenotype within solid tumors was observed in renal cell carcinoma (RCC) patients. In contrast to clear cell (cc) RCC, g1 ILCs were significantly increased in chromophobe (ch) RCC, with most g1 ILCs expressing CD103 and CD49a, indicative of an ieILC1 phenotype [70]. Intriguingly, the ILC1 gene signature generated from the scRNA-Seq data was associated with better survival in chRCC patients but worse survival in ccRCC patients. This finding coincided with decreased expression of GzmA in ieILC1s from ccRCC, as compared to matched conventional NK cells, while in chRCC ieILC1s showed significantly higher GzmA expression, highlighting once more the disparate flavors of g1 ILCs in different tumor entities [70]. A potential role of IL-15 in shaping human g1 ILC phenotype and their anti-tumor functions is advocated by the increased expression of IL-15 in chRCC compared to ccRCC, and the positive correlation between IL-15 levels and ILC1 signature enrichment within the chRCC patient cohort [70]. The differential fates of g1 ILCs and a potential role of IL-15 also became apparent in the scRNA-Seq analysis of Lin^-^ CD56^+^ and/or CD127^+^ cells from tumor and PB samples derived from patients with head and neck squamous cell carcinoma (HNSCC). Pseudotime ordering of cells exhibiting NK cell signatures revealed a developmental trajectory of conventional NK cells from the periphery through an intermediate NK state with type I IFN activation into either *NR4A2* expressing NK cells or highly cytotoxic ieILC1s. This developmental relationship was recapitulated *in vitro* through co-culture of PB NK cells with HNSCC cell lines and IL-15. These experiments showed that both IL-15 and cell-to-cell contact signals delivered by cancer cells were important for the development of immature CD56^bright^ NK cells into highly cytotoxic ieILC1s. As tumor cells produced only small amounts of TGF-β and did not express TGF-β on the surface, the authors propose that in this system additional receptor-ligand interactions contribute to promote tumor-mediated differentiation towards ieILC1s [80]. The dependency of this phenomenon on cell contact emphasizes once more that tumor cells might directly induce a cytotoxic phenotype in g1 ILCs. Intriguingly, both murine ILC1ls in the PyMT model and human Lin^-^CD56^+^CD127^-^CD7^+^CD45RO^+^ cells in CRC are accompanied by a clear enrichment of CD8^+^ ILTKCs with high killing potential, pointing towards an overarching program of innate-like tumor cytotoxicity possibly governed by tumor-derived IL-15 and additional signals [31], [69], [78].

Altogether, similarly diverging g1 ILC phenotypes can be detected in human cancer patient samples, as observed in murine tumor models. In some settings, the TME induces the upregulation of inhibitory receptors/exhaustion molecules, reminiscent of what is observed in exhausted CD8^+^ T cells, while in other settings g1 ILCs display concomitant expression of tissue residency markers and cytotoxic molecules (Fig. 1).

## 7 Conclusions

Taken together the TME affects g1 ILCs in two fundamentally opposing ways – impairment or enhancement of anti-tumor functions. In both cases, TGF-β plays a major role in driving intratumoral differentiation and functional modulation of g1 ILCs. While TGF-β via a non-canonical signaling pathway involving TGF-β-R1 and SMAD4 leads to less cytotoxic NK cells, signaling via TGF-β-R2 seems necessary for the generation of cytotoxic ILC1ls [23], [31], [32], suggesting that different TMEs might promote activation of distinct TGF-β pathways. Further understanding of these signaling pathways will be instrumental for their selective manipulation as a potential future treatment option against different cancers.

Next to TGF-β, IL-15 represents a second crucial signal shaping g1 ILC phenotypes and functions. Exposure to IL-15 and possibly other contact signals directly presented by the tumor favors acquisition of cytotoxic molecules and ILC1 differentiation towards tumor-killing ieILC1s or ILC1ls [21], [31], [32], [80]. Interestingly, IL-15 expressing tumors seem to promote cytotoxicity in a broader range of immune cells, as cytotoxic g1 ILCs were associated with an enrichment of CD8^+^ T cells/ILTKCs, both in the PyMT model as well as in human CRC. These data argue in favor of a cytotoxic module of immune surveillance controlling IL-15-producing tumors [31], [69], [78]. Therefore, manipulation of the TME to induce IL-15 might prove to be a useful strategy for future anti-tumor therapies. Additionally, achieving a better understanding of the differences in g1 ILC behavior in IL-15^+^ and IL-15^-^ tumors can serve to inform distinct treatment choices. In tumors lacking IL-15 production, g1 ILCs appear to be associated with negative outcomes and should be depleted, or manipulated to re-establish their anti-tumor functions. In contrast, in IL-15 producing tumors, enrichment of cytotoxic g1 ILCs could improve therapy outcome.
